# Retraction: Over-expression of microRNA-758 inhibited proliferation, migration, invasion and promoted apoptosis of non-small cell lung cancer cells by negatively regulating HMGB

**DOI:** 10.1042/BSR20180855_RET

**Published:** 2026-02-04

**Authors:** Guo-Hua Zhou, Yi-Yu Lu, Jing-Lian Xie, Zi-Kun Gao, Xiao-Bo Wu, Wei-Shen Yao, Wei-Guang Gu

This article is being retracted from *Bioscience Reports* at the request of the Editor-in-Chief and the Editorial Board. This follows the receipt of a notification from a reader, alerting the Editorial Board to concerns regarding the stylisation and appearance of the Western Blots, as well as the acknowledgment statement. The authors were contacted with regard to the concerns and retraction but did not respond to the Journal's queries or the concerns raised. Given the extent of the issues raised, the Editorial Board stand by the decision to retract the article.

